# A systematic review on the relationship between the built environment and children’s quality of life

**DOI:** 10.21203/rs.3.rs-2828550/v1

**Published:** 2023-04-25

**Authors:** Lisa Hitch, Hanish Kodali, Marc Starvaggi, Katarzyna E Wyka, Terry TK Huang

**Affiliations:** Center for Systems and Community Design and NYU-CUNY Prevention Research Center, Graduate School of Public Health & Health Policy, City University of New York; Center for Systems and Community Design and NYU-CUNY Prevention Research Center, Graduate School of Public Health & Health Policy, City University of New York; Center for Systems and Community Design and NYU-CUNY Prevention Research Center, Graduate School of Public Health & Health Policy, City University of New York; Center for Systems and Community Design and NYU-CUNY Prevention Research Center, Graduate School of Public Health & Health Policy, City University of New York; Center for Systems and Community Design and NYU-CUNY Prevention Research Center, Graduate School of Public Health & Health Policy, City University of New York

**Keywords:** Quality of life, wellbeing, built environment, physical environment, neighborhood, children

## Abstract

**Background:**

Evidence of the effects of the built environment on children has mainly focused on disease outcomes; however, quality of life (QoL) has gained increasing attention as an important health and policy endpoint itself. Research on built environment effects on children’s QoL could inform public health programs and urban planning and design.

**Objective:**

We aimed to review and synthesize the evidence of the relationship between built environment features and children’s QoL.

**Methods:**

Five research databases were searched for quantitative peer-reviewed studies on children between 2–18 years, published in English or German between 2010–2021. Only primary research was considered. Included studies (n=17) were coded and methodologically assessed with the Joanna Briggs Critical Appraisal Checklists, and relevant data were extracted, analyzed, and synthesized, using the following built environment thematic framework: 1) neighborhood natural environment, 2) neighborhood infrastructure, and 3) neighborhood perception.

**Results:**

Green space was positively associated with children’s QoL. Infrastructure yielded inconclusive results across all measured aspects. Overall neighborhood satisfaction was positively correlated with higher QoL but results on perceived environmental safety were mixed.

**Conclusions:**

Most studies are correlational, making it difficult to infer causality. While the positive findings of green space on QoL are consistent, specific features of the built environment show inconsistent results. Overall perception of the built environment, such as neighborhood satisfaction, also shows more robust results compared to perceptions of specific features of the built environment. Due to the heterogeneity of both built environment and QoL measures, consistent definitions of both concepts will help advance this area of research.

## Background

Quality of life (QoL) —a health and wellbeing indicator— has become increasingly important in research on children (1–3). QoL is a multidimensional concept that involves one’s life perception, including physical and mental health, the emotional state, social relationships, environmental features, and cultural values (3–6). As such, QoL is closely linked to wellbeing, a concept focused on three domains: physical, mental, and social health (7). For the purpose of this paper, we consider QoL and wellbeing as interchangeable.

To move beyond traditional health indicators such as mortality and morbidity, QoL introduces a more humanistic element into health research with a focus on an individual’s holistic wellbeing and health (4). QoL measured in children can be used to evaluate interventions, explore facilitatory conditions, inform policies, and support resource allocation based on QoL barriers (8). To date, QoL has been studied in the context of physical activity, social relationships, and mental health and in clinical research on cancer, cardiovascular disease, diabetes, and asthma (3, 4, 7, 9). At the same time, the built environment has also been shown to influence children’s health (10–12). Built environment is defined as the physical surroundings and the perception of these, including land use, cleanliness and aesthetics, and infrastructure (10, 11, 13). However, the role of built environment measures in children’s QoL is not well understood.

Previous research has largely focused on single aspects of the built environment, mainly green space, and their effects on children’s physical and mental health (12, 14). Higher rates of air pollution and noise, greater access to fast-food restaurants, and less availability of and accessibility to green spaces in urban areas have been associated with poorer physical health in cross-sectional studies (10, 11). Physical activity has been associated with active commutes such as walking and cycling in pedestrian-friendly neighborhoods, particularly those with greater amounts of green space, proximity to parks, and quality neighborhood features such as street connectivity and self-reported walkability (10, 11, 13). Perceived safety as it relates to the physical built environment and adequate streetlights have also been related to physical activity (10, 11). Similarly, green space accessibility and use have been shown to improve mental health by reducing stress, promoting resilience, and improving mood (15, 16).

To date, no systematic review has examined the built environment in relation to children’s QoL. As such, we aimed to review the evidence by identifying, evaluating, and synthesizing relevant studies; determining factors that facilitate or impede QoL in children; and exploring how these factors can be considered in programs and urban design.

## Methods

This systematic review was conducted in compliance with the Preferred Reporting Items for Systematic Reviews and Meta-Analyses (PRISMA) (17) and registered in the International Prospective Register of Systematic Reviews (PROSPERO; CRD42021286640).

### Inclusion Criteria

#### 1. Population

Our target population included children aged 2–18 years. Because common QoL questionnaires require the child’s developmental stage to be advanced enough to observe and describe various life dimensions, children < 2 years have been excluded (18).

#### 2. Exposure

The exposure variable consisted of neighborhood built environment measures, defined as the structural aspects of the physical living environment. Since previous literature has indicated the influence of subjectively perceived neighborhood environment on health outcomes, we also included neighborhood perception, and satisfaction (19). Furthermore, since environmental perceptions are specific to the built environment, we expanded the neighborhood perception by concepts of safety as it relates to the environment and traffic. As such, we excluded safety perception of crime as it pertains to the social environment and is thus outside the scope of this paper. The exposure variable was divided into three categories:

1. Neighborhood natural environment: Proximity or quantity of green and blue space, parks, etc., in the context of an urban landscape.
2. Neighborhood infrastructure: Measures of traffic, street connectivity, and availability and accessibility of public transportation, and self-reported outdoor places for play.
3. Neighborhood perception: Subjective neighborhood perception, including overall neighborhood satisfaction, perceived walkability, and environmental safety.

#### 3. Outcome

The outcome consisted of standardized and validated measures of children’s QoL (self- or parent-reported). Due to QoL being closely linked to wellbeing, this systematic review focuses on both concepts, even though only QoL will be used as a term henceforth.

#### 4. Other Inclusion Criteria

Only peer-reviewed quantitative primary research was included. All studies were limited to English and German due to the development of the two largest QoL questionnaires: the KINDL questionnaire (originally developed in German) and the Pediatric Quality of Life Inventory (PedsQL, originally developed in English). To ensure the most recent research, we included studies from January 2010 to December 2022.

#### 5. Exclusion Criteria

Studies on clinical populations and qualitative studies were excluded. To ensure a focus on the neighborhood built environment, we also excluded studies focused on the school or home environment, and studies that assessed safety in terms of interpersonal relations with neighbors or community members (e.g., community trust, social cohesion, or neighbor disputes). Other exclusion criteria included unavailable full-texts, non-human subjects, study protocols, and development, validation or feasibility studies.

### Search Strategy & Data Collection

We applied a 4-step search strategy with the main search occurring between August and September 2021, and two updated searches in January and December 2022. First, we identified keywords through a preliminary search in PubMed. Second, we developed a full search string tailored to the following databases: PubMed, Cochrane Reviews, CINAHL, PsycINFO, Embase, and Web of Science (see Appendix A for tailored search strings). Third, the databases were searched. Fourth, reference lists of all eligible studies for full-text review were screened for additional qualifying studies.

### Study Selection

All search results (n = 6,571) were exported to EndNote and filtered for duplicates. The remaining studies were exported into Excel and LH manually filtered for remaining duplicates. LH screened titles and abstracts of the remaining studies for inclusion (see Appendix B for exclusion details). The remaining 49 studies were divided among three reviewers (LH, MS, HK) for full-text screening. Two reviewers screened each study. In case of a dispute, the third reviewer was consulted, and the case was discussed until a consensus was reached. Thirty-two studies were excluded after full-text screening (see Appendix C for details).

### Data Extraction & Synthesis

The final 17 included studies were divided among the three reviewers and coded based on: research question, exposure/intervention, outcome, study design and setting, population, methods, results, discussion, and limitations. Additionally, methodological quality was assessed with the Joanna Briggs Institute Critical Appraisal Checklist in accordance with study types (20). The following cut-offs were used:

- Poor: >3 items marked with “no” or “undetermined”
- Fair: 2–3 items marked with “no” or “undetermined”
- Good: < 2 items are marked with “no” or “undetermined”

Due to the heterogeneity of built environment aspects and the small sample size of included studies, it was not feasible to conduct a meta-analysis. Each study was then categorized according to a thematic framework developed by the authors based on previous literature: 1) neighborhood natural environment, 2) neighborhood infrastructure, and 3) neighborhood perception. Subsequently, the relationship between built environment and children’s QoL was analyzed, evaluated, and interpreted in the context of other studies within the same category. Some studies intersected categories due to measuring multiple exposures. In addition, this review included studies that utilized both self-reported and objectively measured built environment.

## Results

**Figure 1:**
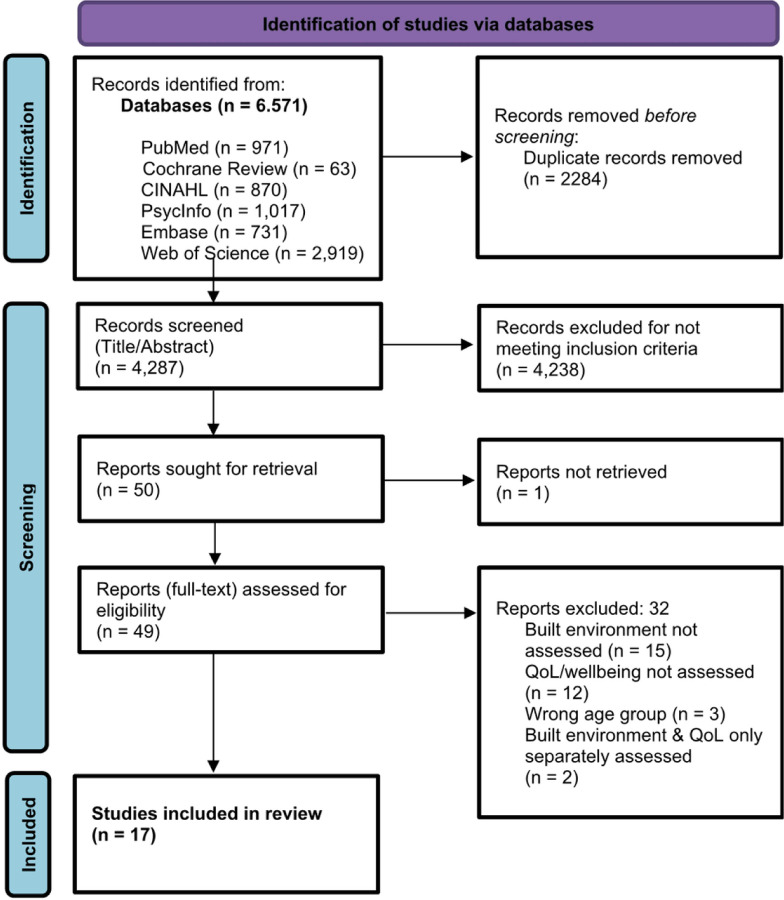
PRISMA Flow Chart

Figure 1 shows this study’s PRISMA flowchart. More detailed exclusion criteria can be found in Supplemental Material S1 and S2.

Table 1 shows a summary of the 17 included studies and their characteristics (21–37).

Study designs ranged from cross-sectional (n = 13) (21–27, 31–34, 36, 37), to longitudinal designs (n = 2) (28, 30), one mixed method study (with a cross-sectional quantitative part) (29), and one intervention design (35). Seventy-five percent (75%) of the studies were published within the prior 5 years. Most studies focused on Europe and North America, whereas four studies focused on South America, Asia, Africa, and Australia (30, 31, 33, 34). The participant ages ranged from 3 to 18 years, with most studies including middle childhood and early adolescence (7–13 years) (21–25, 27, 28, 30–34, 36, 37). Sample sizes ranged from n = 63 to 21,019 participants. More than 80% of studies were of good methodological quality according to the Joanna Briggs Institute Critical Appraisal Checklists (see Supplemental Material S3) (21–29, 32, 34–37).

### Independent variable: Built environment

Studies encompassed a range of objective and subjective built environment measures. Four studies assessed the built environment through objectively measured means only, including landscape spatial patterns and green space quantity utilizing the Geographic Information System (GIS), the Scottish Neighborhood Deprivation Index, street connectivity, facility and playground density, and traffic (21, 24, 25, 32). Eleven studies utilized subjective survey measurements of the built environment including questions regarding neighborhood perception and satisfaction, housing, traffic, environmental safety, public transportation, accessibility and quality of green spaces, playgrounds, open spaces, and spaces for play (23, 26–31, 33, 34, 36, 37). One study combined objectively and subjectively measures of the built environment (22), and another single study utilized an intervention design exposing the participants to green space in the form of small urban parks, larger urban parks, and forest settings (35).

### Dependent variable: Children’s QoL

QoL measurements varied significantly. The Pediatric Quality of Life Inventory (PedsQL) (5), KINDL (6), and KIDSCREEN (38), were the most commonly used scales in seven of the 17 studies. Other measures unique to each study can be found in Table 1.

More than 75% of studies included child-reported QoL measures (21, 23–25, 27, 28, 31–37), whereas 3 studies utilized parent-reported outcomes (26, 29, 30), and one study utilized both self- and parent-reported QoL (22).

### Review findings

**Figure 2:**
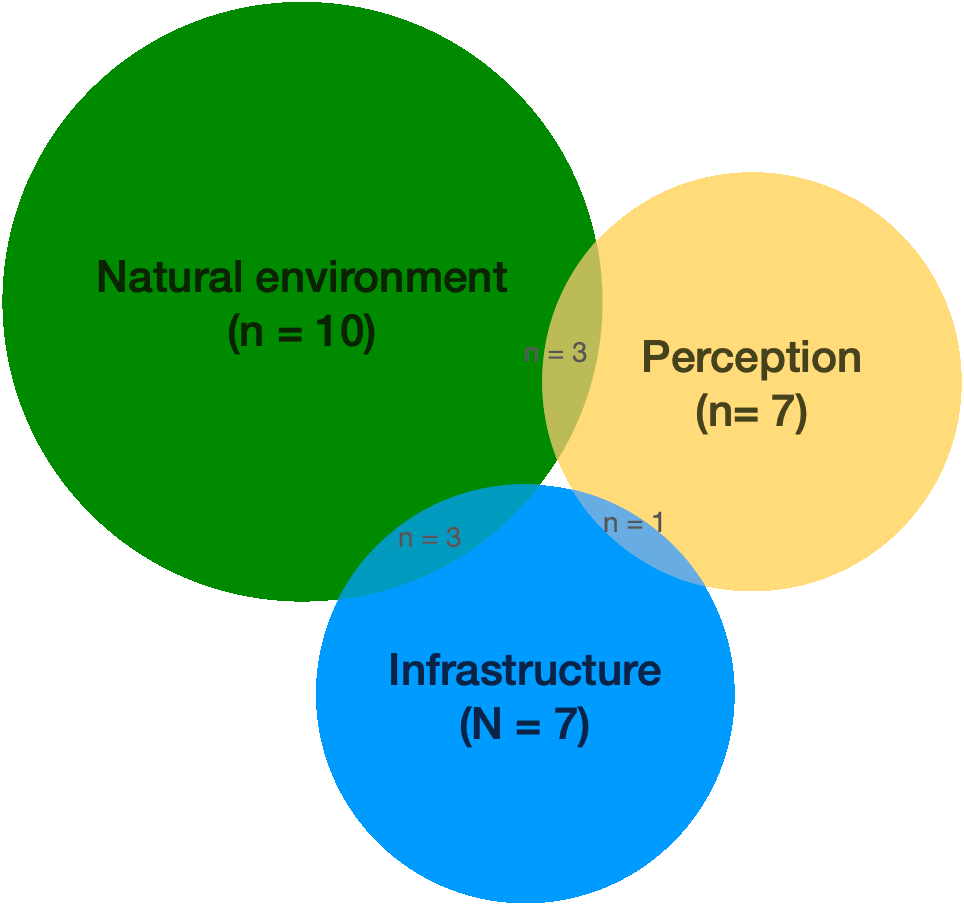
Thematic framework applied to included studies

Figure 2 shows the distribution of studies by the three thematic categories.

### Neighborhood Natural environment and QoL

Ten studies focused on the natural environment with objective measures including number of tree areas, green space quantity, and percentage of green and blue spaces (i.e., bodies of water) in neighborhoods and subjective measures including presence of green, blue or open spaces, and accessibility to these spaces.

On one side, most studies showed a positive relationship between the natural environment and children’s QoL (22, 25, 27–30, 32, 34, 35). More and greater sizes of tree areas or higher proportions of green space within the neighborhood landscape were associated with higher QoL (22, 25, 30, 32). These findings were corroborated by studies utilizing self-reported measures (27–29). On another side, two studies found negative associations between greater distances from one tree area to another and self-reported access to green space and QoL (22, 32). Two other studies did not find significant results for the proportion of green space and children’s QoL (21, 24).

The evidence is mixed regarding blue space. While one study found a negative association of blue space percentage in the neighborhood with children’s QoL (25), another reported a positive relationship (29).

The only intervention study in this systematic review utilized a school lunch break to expose children to either a forest setting or a large or small urban park (35). The authors found a positive relationship between exposure to different kinds (and sizes) of green space and children’s wellbeing (35). The authors also found a dose-response relationship with small urban parks yielding the smallest effects on wellbeing (still significant) compared to indoor lunch breaks and large forest settings yielding the greatest increase in self-reported wellbeing (35).

### Neighborhood Infrastructure and QoL

Seven studies included measures such as street connectivity, walkability, athletic and recreational facility density, and neighborhood deprivation—a measure of neighborhood resource availability and access to employment, education, health, and housing (21, 26, 28, 32–34, 36). The evidence is mixed and inconclusive. Two studies found non-significant results (21, 24). Public transportation access and use by children without adult supervision was negatively associated with children’s QoL (33). Self-reported places to play outside the home also showed mixed findings with one study showing a positive relationship with children’s QoL, and two other studies not confirming these findings due to statistical insignificance (26, 27, 36).

### Neighborhood Perception & QoL

Seven studies included self-reported neighborhood perception (22, 23, 27, 30, 31, 34, 37). Perceived barriers to walking were negatively associated with children’s QoL in one study (22). Broad concepts such as overall neighborhood quality and satisfaction were positively associated with children’s QoL (27, 30, 37). While perceived environmental safety concepts showed a positive relationship with children’s QoL in three studies (23, 31, 34), two other studies found the association to be non-significant (23, 27).

## Discussion

This is the first systematic review on the relationship between the built environment and children’s QoL. Previous research, mostly in adults, has been limited largely to the role of green space in disease outcomes. However, as QoL gains recognition as an important health outcome in its own right, understanding how diverse factors in the neighborhood built environment affect children’s QoL is important to policy and program development and urban design.

Our findings yielded robust evidence for the natural environment and mixed or inconclusive evidence for infrastructure and neighborhood perception. Green space showed a positive relationship with children’s QoL, further supported by a dose-response relationship found in one intervention study. The more green space available in a neighborhood, the higher the QoL among children. This is in line with previous research focused on the benefits of green space in urban environments on mental health, physical activity, and wellbeing across different adult populations (39–41). However, two studies found no significant relationship, indicating measurement errors, small sample sizes, or a homogenous context not providing enough variability in exposure and outcome measures.

While green space is positively associated with children’s QoL in some studies, the importance of open spaces should be highlighted. In Houston (Texas), Hispanic children’s QoL was positively influenced by a greater number of urban forests and by longer distances between these tree patches (22). This highlights the importance of open spaces, including settings with clear edge conditions without dense understories, to provide a sense of safety due to a greater ability to see far, which in turn influences QoL (22). This is in line with a Norwegian study showing a negative relationship between self-reported access to green spaces and children’s QoL in densely populated areas (32). Hence, while urban areas provide more access to parks, these spaces are also associated with traffic, safety concerns, and crowding (32). Thus, the quality of parks and green spaces may have a greater influence on QoL than quantity or accessibility.

Although green spaces are an essential aspect of the built environment, other aspects such as blue spaces are understudied. These have been proposed to be an effect modifier on the relationship between nature and QoL (42). Blue space can contribute to physical activity through access to water sports, and to social health through social gatherings (42). In our review, blue space showed mixed evidence. While blue space was positively associated with children’s QoL in one study, another found the opposite; however, neither study assessed the quality of blue spaces. It is conceivable that cleaner and aesthetically pleasing blue spaces contribute to improved wellbeing, whereas polluted urban rivers or canals may have the opposite effect (25). Furthermore, green and blue spaces are often intertwined with blue spaces being situated within parks, making it difficult to extract the sole effect of blue space on QoL (42).

Our findings showed inconclusive results for neighborhood infrastructure, measured mainly via surveys on select dimensions. Most studies focused on neighborhood deprivation, public transportation accessibility, facility density, street connectivity, and walkability; none of these demonstrated a significant relationship with children’s QoL (21, 24, 32, 33). However, these constructs were researched in isolation and not as a holistic concept, neglecting potentially synergistic effects. Furthermore, self-reported places to play showed inconclusive results with a German study on 6-years olds showing a positive relationship with QoL and two Canadian studies not showing significant results in children 9 years and older (26, 27, 36). Places to play can more directly affect younger children, while other infrastructure constructs measured in other studies may be more relevant to middle childhood, adolescence, and adulthood.

Overall neighborhood satisfaction showed a more positive association with children QoL than narrow constructs such as environmental safety perceptions. This is in line with previous research highlighting that residents in deprived neighborhoods reported lower neighborhood satisfaction even if green spaces and local amenities were evenly distributed compared to less deprived areas (43). This highlights the importance of subjective or experiential measures of neighborhoods even though research has tended to favor objective, GIS-related measures. Furthermore, the disconnect between perception and objective environment may indicate that other factors beyond the built environment influence neighborhood satisfaction and health, such as the social environment and the degree to which a neighborhood is collectivist, which have not been well studied in conjunction with the physical built environment (37).

Notably, cause-and-effect relationships are difficult to determine because of the lack of longitudinal studies. Furthermore, we caution against extrapolating findings from this systematic review across settings. Most studies were conducted in developed countries. Further research is needed to determine how built environment and QoL are related across cultures and countries.

Several challenges have emerged with this review. First, QoL definitions were heterogeneous, with a blurry distinction between related concepts including happiness, life satisfaction, and self-reported physical and mental health, resulting in a wide range of instruments. A more coherent definition of QoL is needed to enhance the comparability of studies. Second, there is also a lack of a coherent definition for built environment measures. Most studies either utilized GIS regarding landscape spatial patterns or study-specific single questions incorporated into a survey. These neglect the multiple dimensions of the built environment and could explain the non-significant findings. The framework applied in this review can serve as a starting point for a more integrated measurement approach.

Finally, we found contradictory results and inconclusive findings within all three categories of our thematic framework; a possible explanation is the difference between accessibility versus actual use of these settings. Actual use of green spaces may have a greater effect on children’s QoL than access alone (24, 44, 45). Green space quality may partially explain this difference. Community-tailored social and physical activity programs, ideally supervised, may be ways to increase park use (46–48). Yet, there is a dearth of intervention studies on the built environment in relation to children’s QoL and more such studies are urgently needed.

## Conclusions

The built environment, especially green and open spaces, and overall neighborhood satisfaction, may play an important role in the QoL or wellbeing of children. Public health programs should integrate with urban design strategies to leverage built environment enhancements to improve children’s health. Additionally, social functions of the built environment, the actual use of these spaces, and subjective experience may play a role in the relationship between the built environment and children’s QoL, suggesting a need for increased attention to social programs within green spaces and ways of improving neighborhood satisfaction. More prospective and intervention studies are warranted to establish causal pathways between the built environment and children’s QoL.

## Supplementary Material

Supplement 1

## Figures and Tables

**Table 1:** Summary and characteristics of included studies

Reference	Thematic framework	Study Design	Study Setting/context	Age range	Sample size	Exposure	Outcome Measurement	Main results
Kim, J. H., et al. (2016) Reference number: (22)	Natural environment & Perception	Cross-sectional	Inner-City Neighborhoods in Houston, Texas, USA	Hispanic children (9–11 years)	92	Objectively measured: landscape spatial patterns Self-reported: neighborhood environmental perception (accessibility, safety, comfort, attractiveness, satisfaction)	Self- and parent reported Pediatric Quality of Life Inventory (PedsQL)(5)	Larger and greater number of tree areas/forests were positively associated with Quality of Life (QoL), as well as longer distances between tree patches. Neighborhood disorder and barriers to walking were negatively associated, but self-reported access to schools and open spaces and existence of parks were positively associated with QoL.
Martin, G., et al. (2021) Reference number: (23)	Perception	Cross-sectional	Schools (Grade 5–8) in Northwestern and Southwestern Ontario, Canada	8–14 years	754–758	Self-reported perceived neighborhood safety (NEWS-Y Survey item, interpersonal safety, traffic safety)	Self-reported Pediatric Quality of Life Inventory (PedsQL)(5)	Traffic safety was not significantly associated with QoL & active school travel. Neighborhood traffic safety was positively correlated with subscales of QoL (emotional and psychosocial functioning), but not with all domains of QoL.
McCracken, D. S., et al. (2016) Reference number: (24)	Natural environment	Cross-sectional	Primary schools in Edinburgh, United Kingdom	8–11 years	287	Objectively measured quantity of green space in neighborhood (ArcGIS, 500m buffer) and level of neighborhood deprivation (SIMD index)	Self-reported Kid-KINDL(6)	Greater greenspace use was associated with better QoL Percentage of greenspace not significantly associated with QoL.
Tillmann, S., et al. (2018) Reference number: (25)	Natural environment	Cross-sectional	Schools (Grade 5–8) in Northwestern and Southwestern Ontario, Canada	8–14 years	851	Objectively measured natural environment: Accessibility to nature, percentage of park/water areas, landscape spatial patterns, Normalized Difference Vegetation Index	Self-reported Pediatric Quality of Life Inventory (PedsQL)(5)	Percentage of park space was positively associated with QoL. Percentage of water/grass/shrubber was negatively associated with QoL I urban areas, but not in rural areas. Lack of clinically significant relationship.
Weigl, K., et al. (2018) Reference number: (26)	Infrastructure	Cross-sectional	Kindergarten to School in Bavaria (Bamberg, Munich, Ingolstadt, Schwandorf, and Günzburg) in Germany	Mean age: 6 years	3,744	Parent-reported environmental factors (including crowded housing, outside places to play, pollution)	Parent-reported Kiddy-KINDL(6)	Positive relationship between place to play outside and QoL.
Wu, X. Y., et al. (2010) Reference number: (27)	Perception, Natural environment	Cross-sectional	Elementary Schools (Grade 5) in Alberta, Canada	10–11 years	3,421	Parent-reported survey: place of residency, neighborhood satisfaction, neighborhood safety, neighborhood playgrounds and parks	Self-reported EuroQoL 5 Dimensions Youth Version (EQ-5D-Y)(49)	Neighborhood satisfaction was positively associated with QoL. No statistically significant relationship between sidewalks/parks or neighborhood safety and QoL.
Mastorci, F., et al. (2021) Reference number: (28)	Infrastructure, Natural environment	Longitudinal	Middle Schools in Central and Northern Italy (Tuscane, Liguria Friuli Venezia Giulia)	10–14 years	1,289	Self-reported online questionnaire on environment and housing situation (including presence or absence of green spaces or terraces)	Self-reported KIDSCREEN-52(38)	Having green space o terraces is associated with better QoL. Living in city and not having green space associated with reduced physical health domain of QoL
Nagata et al. (2021) Reference number: (29)	Natural environment	Mixed Methods	Parks in Lower Manhattan, New York, New York United States	3–13 years	174	Parent-reported questionnaire on importance of urban farm, frequency of visits to green space, time spent in green space, and proximity to green space	Parent-reported Patient-Reported Outcomes Measurement Information System’s Positive Affect and Life Satisfaction scales(50)	Access to urban farms positively associated with QoL. Residential proximity to blue space associated with better QoL.
Feng et al. (2017) Reference number: (30)	Natural environment, Perception	Longitudinal	Neighborhoods in Wollongong, New South Wales, Australia	4–5 years and 12–13 years	4,968	Objectively measured: Greenspace quantity (percentage of land use) Parent-reported quality of parks using a Likert scale	Parent-reported Strengths and Difficulties Questionnaire (SDQ)(51)	Dose-response relationship was found: the more green space and the better quality of green space the better QoL. But plateau effect: gains i QoL appeared to top out for participants with 21–40% of the residential land-use designated as green space
González-Carrasco et al. (2019) Reference number: (31)	Perception	Cross-sectional	Neighborhoods in Spain, Algeria, South Africa, Israel	< 13 years	9,262	Self-reported questionnaire on neighborhood satisfaction and perceived safety	Self-reported overall satisfaction with life, Students’ Life Satisfaction Scale (SLSS; 5 instead of 11 items) and Brief Multidimensional Students’ Life Satisfaction Scale (BMSLSS) (5 items instead of 40 items)(52, 53)	Higher satisfaction with safety associated with higher QoL.
Nordbø et al. (2020) Reference number: (32)	Infrastructure, Natural environment	Cross-sectional	Densely populated, urban neighborhood in Norway	8 years	21,019	Objectively measured built environment features including number of facilities/amenities, number of playgrounds, area of green space, and access to/presence of park within buffer	Self-reported Short Mood & Feelings Questionnaire (SMFQ)(54)	Organized activities mediate the relationship between green space, access to parks and greater QoL Surprising finding: Greater access to parks was negatively correlated with child wellbeing. Greater total green space was associated with greater QoL
de Macêdo et al. (2021) Reference number: (33)	Infrastructure	Cross-sectional	Commute between home and school in state capitals in Brazil (Curitiba, Florianopolis, Porto Alegre, Rio de Janeiro, Sao Paulo)	9–14 years	1,787	Self-reported questionnaire on public transportation, active commute with or without supervision, and surrounding streets/street connectivity	Self-reported Children’s World International Survey of Children’s Well-Being (ISCWeB) (55)	Playing in streets/parks associated higher QoL going to/from school with adult was associated higher QoL using public transportation without adult was associated with lower QoL.
Lee & Yoo (2015) Reference number: (34)	Infrastructure, Perception	Cross-sectional	Urban neighborhood across 11 countries (Algeria, Brazil, Chile, England, Israel, Romania, South Africa, South Korea, Spain, Uganda, United States)	12 years	12,077	Self-reported survey on community factors including access to areas to play and neighborhood safety	Self-reported General Domain Satisfaction Index (GDSI)(56)	Community factors explain 7% of child’s QoL with more places to play outside and higher perceived safety to walk around in neighborhood associated with greater QoL. Country-specific variation, but for each country on their own, these factors remain significant for child’s QoL.
de Bont et al. (2021) Reference number: (21)	Natural environment, infrastructure	Cross-sectional	Primary Schools in Sabadell, Spain	9–12 years	2,213	Objectively measured, including green space availability and accessibility, street connectivity, facility density, walkability, road traffic (traffic load, traffic density, traffic noise)	Self-reported KIDSCREEN-27(38)	Different built environment clusters had no impact on QoL but were associated with obesity which could be a mediator to QoL. School or social environment may play a role in explaining this relationship.
Wallner et al. (2018) Reference number: (35)	Natural environment	Intervention study	Urban parks in Vienna, Austria	16–18 years	64	Intervention: Exposure during lunch break to either a) small urban park, b) large urban park, or c) forest setting	Self-reported Self-condition scale by Nitsch(57)	Significant differences between time points on QoL. QoL was highest after 1 hour (before leaving). Forest settings consistently (across a time points) exceeded the results from small and large urban parks regarding QoL.
Mitra et al. (2021) Reference number: (36)	Infrastructure	Cross-sectional	Online survey targeting urban neighborhood in Canada	9–15 years	800	Self-reported physical environment including places to play, access to park or other shared outdoor space	Self-reported adopted Russell’s theorization of psychological construct of emotions(58)	Not enough places to play both indoor and outdoor, were associated with reduced QoL. Social component relevant, places to socialize seem to be more important than just places to play. Physical activity was positively correlated with QoL.
Forrester et al. (2022) Reference number: (37)	Perception	Cross-sectional	Urban elementary schools in a Mid-Atlantic state in the United States	Mean age: 9.32 years	63	Self-reported neighborhood quality, neighborhood satisfaction, and places to play in neighborhood	Self-reported Overall Life Satisfaction (OLS), Student Life Satisfaction (SLSS), and domain-specific Personal Wellbeing Index for School Children (PWI-SC)(52, 55, 59)	Neighborhood quality and neighborhood satisfaction was significantly associated with Personal Wellbeing and Student Life Satisfaction. Neighborhood quality accounted for 17.4% of variance in Student Life Satisfaction.

* Methodological quality was assessed with the Joanna Briggs Institute Critical Appraisal Checklists. The proper checklist was chosen according to study type.

<2 items marked as “no” or “undetermined” qualified for good methodological quality, 2–3 items marked as “no” or “undetermined” qualified as fair methodological quality, and > 3 items marked as “no” or “undetermined” qualified as poor methodological quality.

## Appendices

Appendices B and C are not available with this version.

## Abbreviations

BMSLSS: Brief Multidimensional Students’ Life Satisfaction Scale
EQ-5D-Y: EuroQoL 5 Dimensions Youth Version
GDSI: General Domain Satisfaction Index
GIS: Geographic Information System
ISCWeB: Children’s World International Survey of Children’s Well-Being
OLS: Overall Life Satisfaction
PedsQL: Pediatric Quality of Life Inventory
PRISMA: Preferred Reporting Items for Systematic Reviews and Meta-Analyses
PROSPERO: International Prospective Register of Systematic Reviews
PWI-SC: Personal Wellbeing Index for School Children
QOL: Quality of Life
SDQ: Strengths and Difficulties Questionnaire
SLSS: Students’ Life Satisfaction Scale
SMFQ: Self-reported Short Mood & Feelings Questionnaire
