# Supplementary material for: A systematic review on the relationship between the built environment and children’s quality of life
Source: Res Sq. 2023 Apr 25:rs.3.rs-2828550. Preprint. [Version 1] doi: 10.21203/rs.3.rs-2828550/v1 (PMC10168438; doi:10.21203/rs.3.rs-2828550/v1)
Supplement: Supplement 1 [file NIHPPRS2828550V1-supplement-1.pdf]

Supplementary Files

This is a list of supplementary files associated with this preprint. Click to download.

- 041723S1BMCSystematicReviewBuiltEnvironmentQoL.docx
- 041723S2BMCSystematicReviewBuiltEnvironmentQoL.docx
- 041723S3SystematicReviewBuiltEnvironmentQoL.docx
- Appendices.docx
